# Identification of dual *STRN-NTRK2* rearrangements in a high grade sarcoma, with good clinical response to first-line larotrectinib therapy

**DOI:** 10.1186/s13000-023-01400-1

**Published:** 2023-10-21

**Authors:** Ruihe Lin, Atrayee Basu Mallick, Zi-Xuan Wang, Scot Andrew Brown, Bo Lu, Wei Jiang

**Affiliations:** 1 Department of Pathology and Genomic Medicine, Thomas Jefferson University Hospital, Philadelphia, PA USA; 2 Department of Medical Oncology, Thomas Jefferson University Hospital, Philadelphia, PA USA; 3 Orthopaedic Surgery, Rothman Orthopaedic Institute, Thomas Jefferson University Hospital, Philadelphia, PA USA; 4 Radiation Oncology, University of Missouri-Columbia, Columbia, MO USA

**Keywords:** *STRN-NTRK2* fusion, Sarcoma, Next-generation sequencing, Larotrectinib

## Abstract

**Background:**

Among the three *NTRK* genes, *NTRK2* possesses a tremendous structural complexity and involves tumorigenesis of several types of tumors. To date, only *STRN* and *RBPMS* are identified in the fusion with *NTRK2* in adult soft tissue tumors. More recently, the highly selective Trk tyrosine kinases inhibitors, including larotrectinib and entrectinib, have shown significant efficacy for treating tumors harboring *NTRK* fusions and were approved by FDA.

**Case presentation:**

We report a case of sarcoma in a 35-year-old female harboring two *STRN*-*NTRK2* gene fusions, with a good clinical response to first-line larotrectinib treatment. Core biopsy of the 16.5 cm gluteal mass showed a high-grade mesenchymal neoplasm with features reminiscent of a solitary fibrous tumor, but negative for STAT6. In-house next-generation sequencing gene fusion panel showed two in-frame *STRN-NTRK2* fusions, which contain the same 5’ partner sequence (exon 1–3) of *STRN*, and the 3’ fusion partner starting from either the exon 15 or the exon 16 of *NTRK2*. Due to the large size and location of the tumor, first-line neoadjuvant therapy with larotrectinib was initiated. The patient has an excellent clinical response with an 83% tumor size reduction by imaging. The tumor was subsequently completely resected. After 130 days, larotrectinib was reinitiated for lung metastasis (up to 7 cm), and a complete resolution was achieved. When compared with *NTRK1* and *NTRK3*, *NTRK2* fusions are the least common. Of note, the only other report in the literature on *NRTK2* fusion-positive sarcoma also showed solitary fibrous tumor (SFT)-like morphology, and the patient responded well to larotrectinib as the second line adjuvant therapy.

**Conclusions:**

In conclusion, the identification of *NTRK2* fusions in patients with soft tissue tumors could significantly improve the clinical outcome through selective *NTRK* inhibitor therapy, especially in the first-line setting. Prompt RNA-based NGS testing at initial diagnosis may benefit these patients. Our case is among the first few in the literature on *NTRK2* fusion sarcoma with first-line larotrectinib therapy in the primary and metastatic setting, with good clinical response and minimal side effects.

## Background

TrkA, TrkB, and TrkC are receptor tyrosine kinases encoded by *NTRK1-3* genes. They are activated by binding with neurotrophins [1–3]. The Trk proteins share highly homologous sequence and structural features, including an extracellular region, a transmembrane region, and an intracellular tyrosine kinase domain, which upon activation, transduces the downstream signaling via MAPK, PI3K, and PKC pathways [4]. Intrachromosomal or interchromosomal *NTRK* gene fusion is the most noticeable underlying mechanism of oncogenesis. The fusion protein contains in-frame N-terminal amino acids from the fusion partner and the C-terminal amino acids containing the tyrosine kinase domain of the Trk receptor, resulting in a constitutively active chimeric kinase [1–7]. More than 80 genes have been identified as partners in the fusion to *NTRK* genes [8].

Among the three *NTRK* genes, *NTRK2* possesses a tremendous structural complexity and involves oncogenesis of several types of tumors. To date, there are approximately 42 partner genes involving *NTRK2* rearrangement [2, 3, 5, 8–12], and only *STRN* and *RBPMS* are identified in the fusion with *NTRK2* in adult soft tissue tumors [3] (Table 1).

**Table 1:** Review of reported NTRK2 fusion tumors with different partner genes

ID	Partner genes	Tumor types (Refs)	Tumor location(s)
1 (including current case)	STRN	Sarcoma (current case, 14), Glioneuronal tumor (12)	Gluteal / Pelvic(current case), retroperitoneal, CNS
2	RBPMS	Sarcoma (3)	soft tissue
3	WWOX	Sarcoma (9)	Uterine
4	GNAQ	Sarcoma (3)	Bone
5	DAB2IP	Squamous cell carcinoma, Breast carcinoma, Lung adenocarcinoma (3)	Colorectal, Breast, Lung
6	TRAF2	Melanoma (3)	Skin
7	PAN3	Squamous cell carcinoma (3)	Head & neck
8	ETV6	AML (3)	Hematologic
9	SQSTM1	Glioma, Lung adenocarcinoma (1)	CNS, Lung
10	TRIM24	Ganglioglioma, Lung adenocarcinoma (10)	CNS, Lung
11	BCR	Glioma, Gangliocytoma (1)	CNS
12	C2orf44	Glioma (10)	CNS
13	KANK1	Glioma (10)	CNS
14	AFAP1	Glioma (3)	CNS
15	AGBL4	Glioma (3)	CNS
16	GKAP1	Glioma (1)	CNS
17	QKI	Glioma (10)	CNS
18	KCTD8	Glioma (1)	CNS
19	NACC2	Glioma (3)	CNS
20	NOS1AP	Glioma (1)	CNS
21	PRKAR2A	Glioma (1)	CNS
22	VCL	Glioma (3)	CNS
23	VCAN	Glioma (1)	CNS
24	TBC1D2	Glioma (1)	CNS
25	KCTD16	Ganglioglioma (10)	CNS
26	STRN3	Ganglioglioma (11)	CNS
27	SPECC1L	Glioneuronal tumor (10)	CNS
28	WNK2	Glioneuronal tumor (11)	CNS
29	TLE4	Ganglioglioma (3)	CNS
30	ACO1	Not specified (8)	Not specified
31	CTDSP2	Not specified (8)	Not specified
32	DENND1A	Not specified (8)	Not specified
33	FAM117B	Not specified (8)	Not specified
34	NAV1	Not specified (3)	Not specified
35	NOD1	Not specified (8)	Not specified
36	PAIP1	Not specified (8)	Not specified
37	PCSK5	Not specified (8)	Not specified
38	PPP6R3	Not specified (8)	Not specified
39	PRRX1	Not specified (8)	Not specified
40	SLMAP	Not specified (3)	Not specified
41	THADA	Not specified (8)	Not specified
42	TRIP13	Not specified (8)	Not specified

More recently, the highly selective Trk tyrosine kinases inhibitors, including larotrectinib and entrectinib, have shown significant efficacy for treating tumors harboring *NTRK* fusions and were approved by FDA [2, 7, 13, 14].

Herein, we report the identification of *NTRK2* gene rearrangement by initial RNA-based NGS, with the efficacy of larotrectinib treatment in an adult patient with a sarcoma harboring *STRN-NTRK2* fusions. The results demonstrate the importance of the identification of *NTRK* alteration in soft tissue sarcoma, and the urgent need for mechanistic study for resistance to targeted therapy in *NTRK2* fusion sarcoma.

## Case presentation

**Fig. 1:**
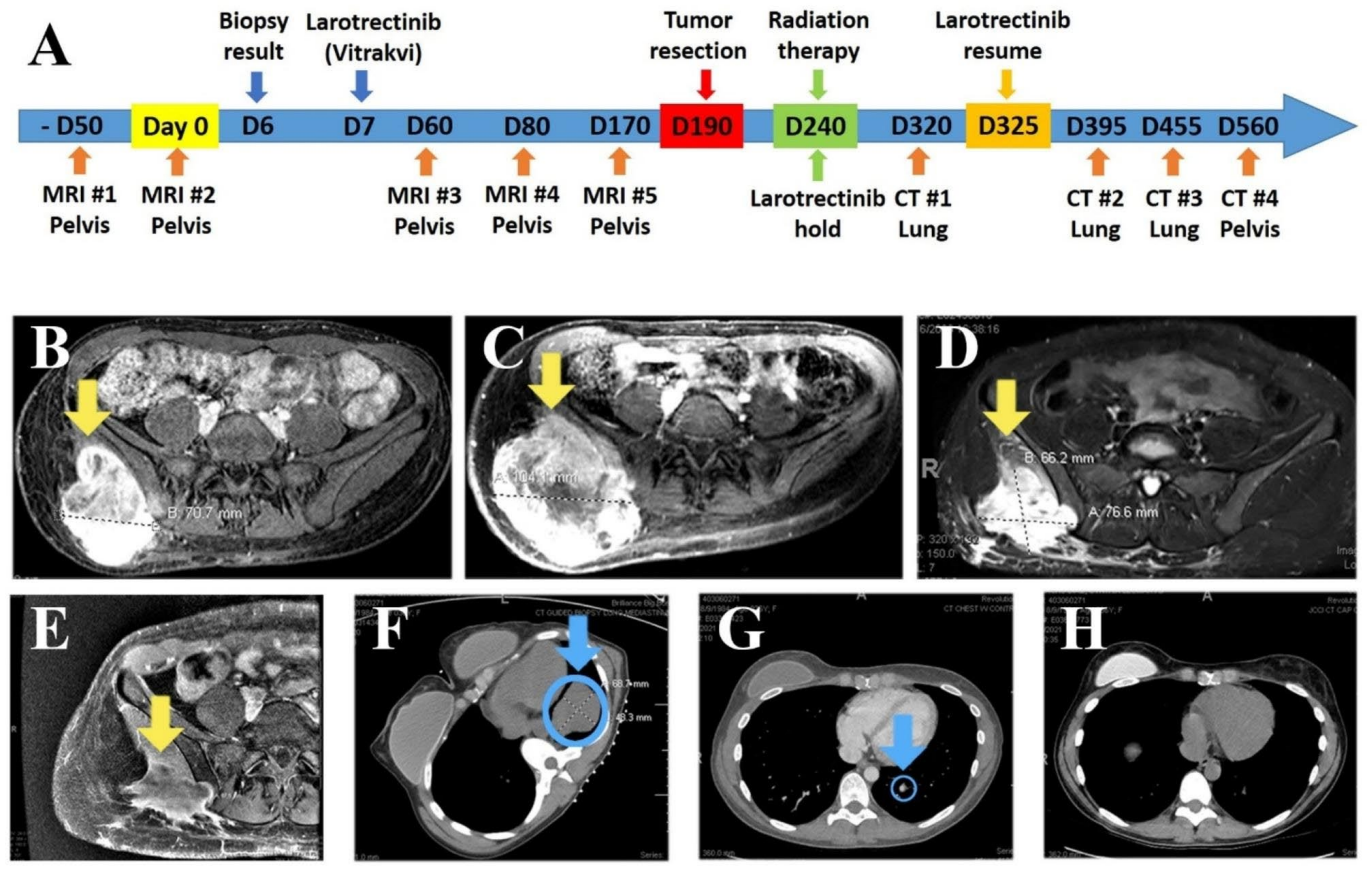
Clinical timeline (**A**) and imaging (**B-E**). Before treatment (B: - Day 50 & C: Day 0), a heterogenous mass (yellow arrows in B-E) centers in the right gluteus medius and maximus muscles with extension into the right paraspinal musculature, and invasion of the right posterior iliac bone. Imaging on post-treatment Day 60 (D) and Day 170 (E) show significant decrease in tumor size (MRI#4 of Day 80 is not shown here). 130 days after surgery, a 6.9 cm solid mass in the lower lobe of left lung is biopsied and proven to be a metastatic lesion (**F**, Day 320, blue arrow with circle), with near complete resolution after the resumption of Larotretinib treatment (**G**: Day 455, blue arrow with circle & **H**: Day 560)

A 35-year-old woman presented to our oncology clinic for a second opinion for a right gluteal sarcoma initially diagnosed outside the continental United States. She complained of nausea, fever, and worsening pain and numbness form the affected area. The mass had been increasing in size. MRI showed a large, heterogeneous, T2 hyperintense, vascular, and partially necrotic and enhancing mass, centered in the right gluteus medius and maximus muscles, with extension into the right paraspinal musculature, and invasion of the right posterior iliac bone. The initial MRI (50 days ago) showed the mass measuring 10.2 × 9.7 × 7.0 cm (Fig. 1B), and measured 16.5 × 12.9 × 10.4 cm (AP x TV x CC) (Fig. 1C) at first encounter at our institution. PET scan showed no metastasis.

**Fig. 2:**
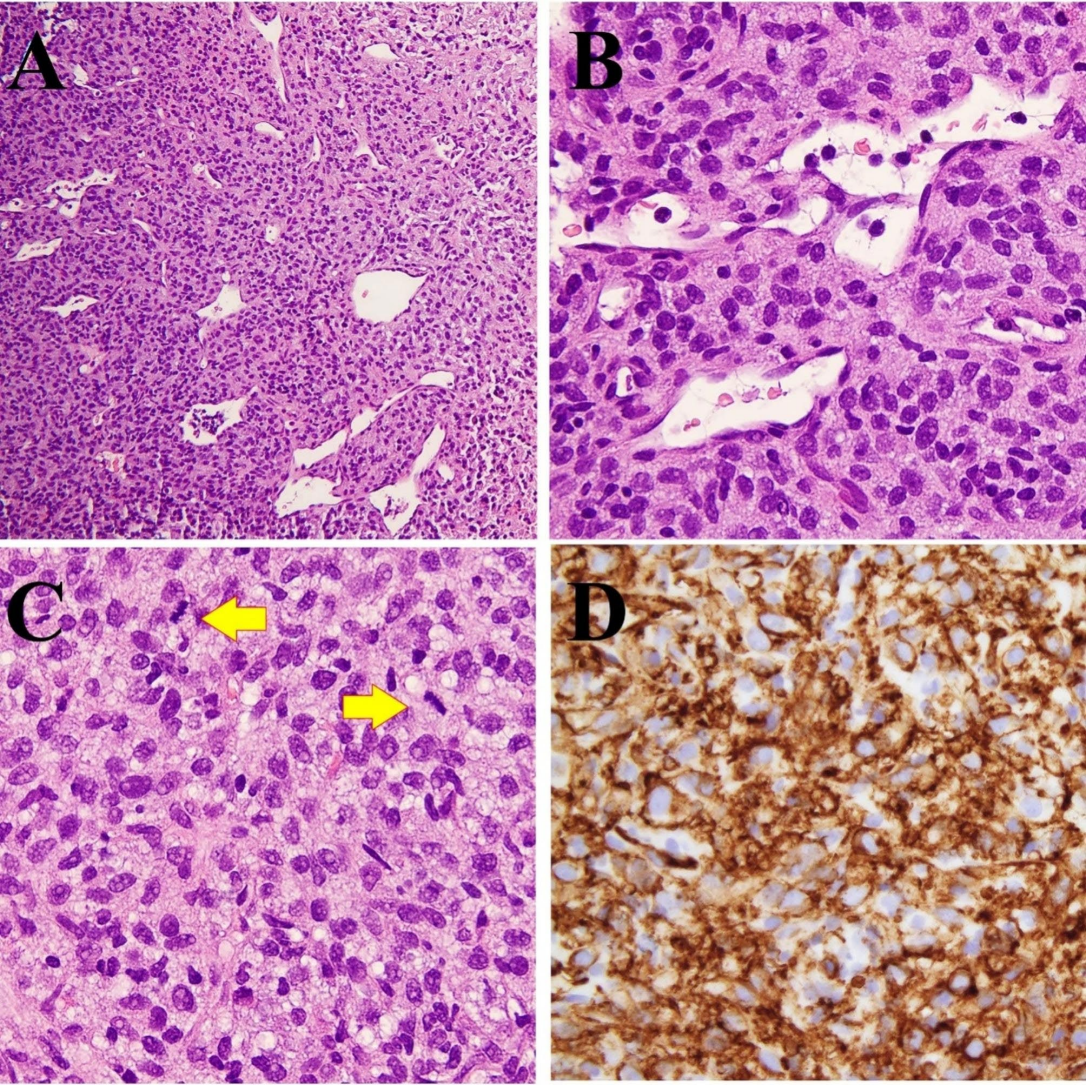
Pathology of the core biopsy. H & E sections show a highly cellular mesenchymal neoplasm of round/ovoid cells with eosinophilic cytoplasm, round to ovoid nuclei and mostly inconspicuous nucleoli without significant nuclear pleomorphism. The tumor is rich in the vasculature with staghorn-type vessels (A & B), and brisk mitotic activity (C, arrows). Tumor is positive for CD34 (D). Magnifications: A, 100X; B-D, 400X

A core biopsy was performed and showed a highly cellular mesenchymal neoplasm consisting of round/ovoid cells, with eosinophilic cytoplasm, round to ovoid nuclei with mostly inconspicuous nucleoli, and no significant nuclear pleomorphism (Fig. 2A and B). The tumor was rich in vasculature, with focal staghorn-type vessels (Fig. 2A). Brisk mitotic activity (21 per 10 high-power fields) and tumor necrosis were identified (Fig. 2C). Histologically, the tumor was reminiscent of a malignant solitary fibrous tumor (SFT). It was diffusely positive for CD34 (Fig. 2D), but negative for STAT6 (data not shown). The tumor was also positive for FLI1 (patchy), TLE1 (patchy), and CD99 (focal and weak), and was negative for AE1/AE3, S100, SOX10, ASMA, desmin, ERG, and CD31. LCA stain highlighted the intermixed inflammatory cells (data not shown).

**Fig. 3:**
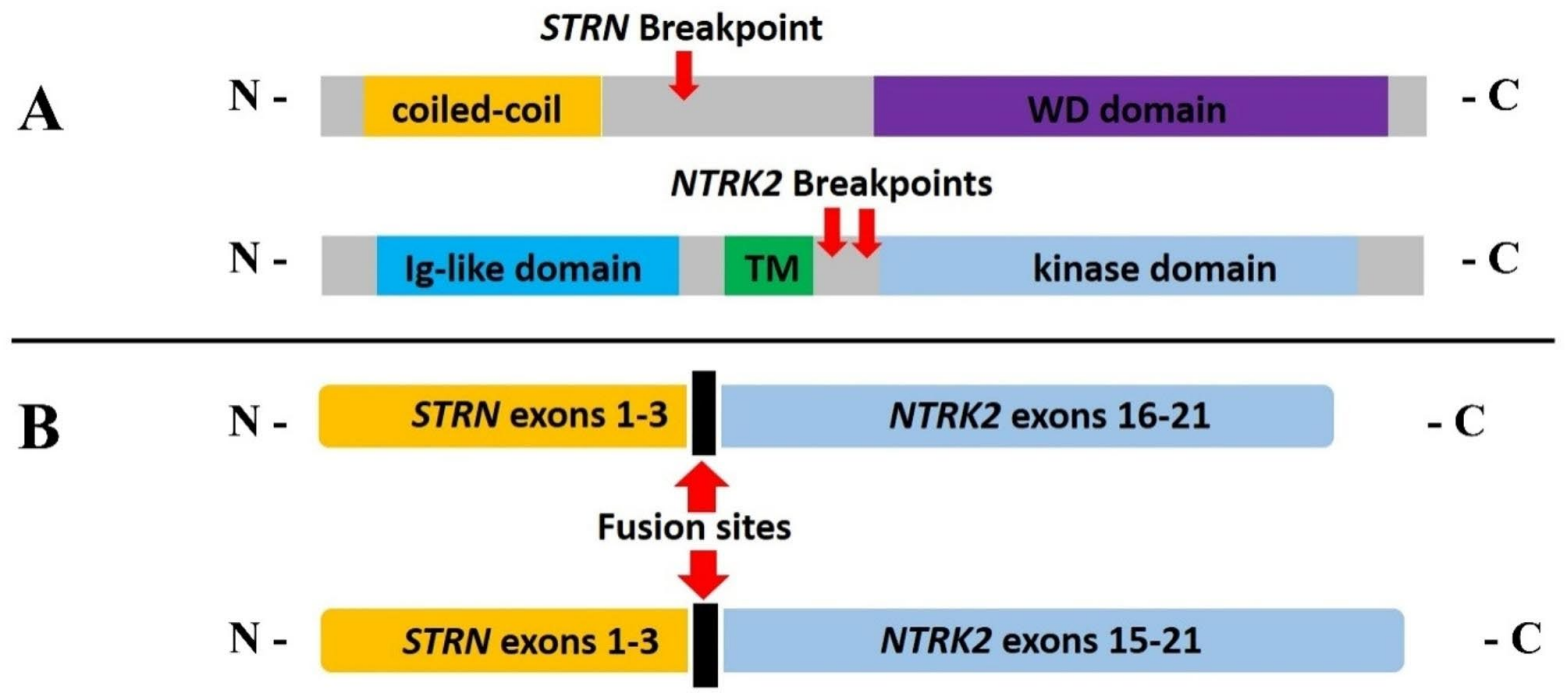
Molecular characterization of tumor sample by NGS. Two in-frame *STRN-NTRK2* fusions with same 5’ partner sequence (exons 1–3) of *STRN* gene identified by NGS.

In-house RNA-based NGS gene fusion panel analysis (Archer FusionPlex™ Comprehensive Thyroid and Lung Panel, 18 gene fusion panel) was performed, and two concurrent *STRN-NTRK2* fusions were identified. The NGS data showed that both fusions were in-frame and located at the exon-exon boundary. Both fusion RNAs had the same 5’ partner sequence (exon 1–3) of the *STRN* gene (chr2, NM_003162.3, breakpoint: 37,143,221, Fig. 3A and B). One of the 3’ fusion sequence started from the exon 15 of *NTRK2* (chr9, NM_006180.4, breakpoint: 87,475,955, Fig. 3A), and the other 3’ fusion sequence began from exon 16 of *NTRK2* (chr9, NM_006180.4, breakpoint: 87,482,158, Fig. 3B). Of note, the fusion product containing exon 1–3 of *STRN* and exon 16–21 of *NTRK2* was the same as the one which was previously identified as an undifferentiated sarcoma in a pediatric patient [14]. The second fusion pattern starting from exon 15 of the *NTRK2* had been identified in a ganglioglioma harboring *TLE4-NTRK2* fusion.

Due to the location and extent of the tumor, it was deemed unresectable or would require a morbid upfront surgery, i.e., hemipelvectomy. Given the NGS findings, multidisciplinary tumor board was held, and the patient was started on the selective *NTRK* inhibitor, Larotrectinib, at 100 mg, BID, as the first-line therapy. The patient noticed a quick amelioration of tumor-related pain and was able to discontinue all pain medicines (previously on 20 mg oxycontin plus 10 mg oxycodone immediate release every 6 h as needed). The patient also reported a significant shrinkage of the tumor after the initial 7-day treatment, and it continued to improve in the following 45 days. On post-treatment day 50, MRI was repeated and showed that the tumor had significantly decreased in size to 6.6 × 7.7 × 7.4 cm (Fig. 1D), approximately 83% reduction compared with the tumor size before treatment. The previously noted focus of enhancement in the right paraspinal musculature was not present in this imaging.

The patient was followed up closely, and MRI was repeated on post-treatment day 90 and day 170. The size of tumor remained stable during this interval (Fig. 1E, day 90: 6.3 × 7.4 × 5.8 cm; day 170: 6.0 × 7.1 × 5.1 cm). During the later period of treatment, the patient started to feel a few nodules around the original tumor expanding with new significant pain. Metastatic workup showed no metastases, and on post-treatment day 190, the patient underwent radical resection of right gluteal soft tissue sarcoma and right ilium, which was uneventful.

The resection specimen revealed a 6.1 × 7.5 × 3.6 cm ill-defined, heterogeneous, fleshy, solid and necrotic mass with a tan-pink cut surface. Histologically, the tumor showed high-grade undifferentiated sarcoma with treatment effects (granulation tissue, myxoid change, fibrosis, foamy macrophages, and necrosis (50% of tumor volume)). It focally invaded into the ilium. Resection margins are free of tumor.

After wound healing was completed, the patient was started on adjuvant radiation therapy (a total of 5000 cgy) given this being a high grade tumor > 10 cm. Larotrectinib was held during adjuvant radiation due to a lack of data on concurrent radiation therapy. Within six weeks after completion of radiation, while still off larotrectenib, patient presented with chest pain and shortness of breath, and chest tightness. Imaging studies showed multiple new large lung nodules. A biopsy of one of the nodules showed metastatic sarcoma. The in-house NGS gene fusion panel (Archer FusionPlex™ Pan Solid Tumor v2 panel, ArcherDX/Invitae, 99 gene panel) showed persistent *NTRK2* fusions. The patient was then restarted on palliative larotrectenib, and the symptoms improved within a few days. CAT scan repeated 60 days after initiation of palliative larotrectenib showed complete resolution of her lung metastases. The patient continued larotrectinib with response for 22 months before developing symptomatic right parietal lobe and leptomeningeal disease, and biopsy showed metastatic sarcoma with fusion panel again positive for *NTRK2-STRN* fusions. Imaging studies to evaluate status of disease showed persistent extracranial response to larotrectenib. Patient was started on compassionate use of second generation NTRK inhibitor PBI-200 with known blood brain barrier penetration. The brain metastasis biopsy was sent for outside laboratory NGS to look for mutations. However, none of the known on-target resistant mutations of TRKB, including solvent front mutations (e.g. TRKB^G639R^), gate keeper residue mutation (TRKB^F633L^), xDFG motif mutation (TRKB^G667C^) [15], and none of the known off-target mutations (e.g., *MET* amplification, *BRAF*^V600E^ mutation, and *KARS* mutations) were identified in this case [15]. Patient had disease control for 3 months on the second generation *NTRK* inhibitor with eventual worsening of intracranial disease and succumbed to her disease.

## Discussion and conclusions

*NTRK* fusions are identified in 0.31% of adult tumors and 0.34% of pediatric tumors. The most common gene was *NTRK3* (0.16% of adult tumors), followed by *NTRK1* (0.14% of pediatric tumors), with *NTRK2* being the least common (0.06% of adult tumors) [5]. The most commonly seen tumor types driven by *NTRK* gene fusion are mammary analog secretory carcinoma, secretory breast carcinoma, and infantile fibrosarcoma (*ETV6-NTRK3* fusions, 70–91%) [5]. Other tumor types with very low incidence (< 2%) include thyroid cancer, breast carcinoma, non-small cell lung cancer, colorectal cancer, melanoma, brain tumors, and sarcoma [5].

Among all the tumors harboring *NTRK* rearrangements, soft tissue tumors are attracting more diagnosis-oriented attention due to the equivocal histologic findings and available targeted therapy. Selective Trk inhibitors such as larotrectinib and entrectinib emerged as the treatment for tumors harboring *NTRK* fusions with FDA approval [2, 7, 13, 14]. However, the complexity of the *NTRK2* gene structure, the diversity of fusion partners, the rarity of the clinical cases, and the limited availability of the detection modalities make the diagnosis and treatment more difficult.

For detection of *NTRK* fusions, several methods are available: immunohistochemistry (IHC, pan-TRK antibody), fluorescence in situ hybridization (FISH), DNA-based NGS, and RNA-based NGS sequencing [16]. In the largest study to date (33,997 cancer cases), DNA-based sequencing showed an overall sensitivity of 81.1% (60/74 cases) and specificity of 99.9% for detection of *NTRK* fusions, when compared to RNA-based sequencing [17]. More specifically, the sensitivity for DNA-based NGS to detect *NTRK1* fusion is 96.8% (30/31 cases), 0% for *NTRK2* fusion (0/4 cases), and 76.9% for *NTRK3* fusions (30/39 cases). For IHC, an overall sensitivity is 87.9%, and specificity is only 81.1% [17]. In fact, for our patient, multiple specimens were also sent to outside commercial laboratories for comprehensive sequencing, and the *NTRK2* fusions were missed twice by two different commercial laboratories using DNA-based NGS methods. Both of the labs repeated the testing using RNA-based NGS, and detected the *NTRK2* fusion. Based on ours and others’ experiences, RNA-based sequencing appears to be the optimal way to identify *NTRK* fusions, especially *NTRK2* fusions, because the splicing out of introns simplifies the technical requirements of adequate coverage, and detection of RNA-level fusions provides direct evidence of functional transcription, therefore, should be the test of choice when possible.

The results of *STRN-NTRK2* fusion from our case show some common as well as unique features when compared with previously reported cases. First, most of these fusion proteins contain the C terminal fragments translated from either exon 15 or exon 16 of *NTRK*. The breakpoint of *STRN* is a recurrent site in various tumors. The product from *STRN* contributes a coiled-coil domain for potential oligomerization. These findings lead to the recognition of these breakpoints as hot spots as well as a common fusion pattern of these two genes in soft tissue tumors. Second, the incidence of *STRN-NTRK2* fusion might be underestimated in soft tissue tumors if the RNA-based NGS panel is not used in the clinical setting. Third, the tumor in our case showed SFT-like histology, and *NTRK* tumors should enter the differential diagnosis of SFT-like tumors with negative STAT6. Finally, the initial and prolonged good clinical response to TRK inhibitor therapy (primary and extracranial metastases), and the ultimate resistance in cranial disease highlighted the urgency for mechanistic study as well as development of newer and better drugs.

In conclusion, the identification of *NTRK2* fusions in patients with soft tissue tumors could significantly improve the clinical outcome through selective *NTRK* inhibitor therapy, especially in the first-line setting. Larotrectenib is very effective in treating either primary or metastatic tumors harboring *NTRK* rearrangement. Due to the lack of specific morphologic pattern and IHC profile, prompt RNA-based NGS testing at initial diagnosis may benefit these patients. However, with the use of TRK inhibitors for about 3 years now, we have started to see development of resistance in these patients to first-line TRK inhibitors, and research is urgently needed to elucidate mechanism of resistance, especially in the *NTRK2* fusion tumors, to develop better 2nd and 3rd generation *NTRK* inhibitors and alternative treatment regimens.

## Data Availability

The data of the present study are available from the corresponding author upon reasonable request.

## Abbreviations

NTRK: Neurotrophic Tyrosine Receptor Kinase
FDA: The United States Food and Drug Administration
IHC: Immunohistochemistry
FISH: Fluorescence in situ hybridization
NGS: Next-Generation Sequencing
SFT: Solitary fibrous tumor
MRI: Magnetic resonance imaging
CAT: Computed tomography
PET: Positron emission tomography
AP: Anterior-Posterior
TV: Transverse
CC: Cranio-Caudal
BID: Bis in die (2 times a day)
MAPK: Mitogen-activated protein kinase
PI3K: Phosphoinositide 3-kinases
PKC: Protein kinase C
